# PSCA expression is associated with favorable tumor features and reduced PSA recurrence in operated prostate cancer

**DOI:** 10.1186/s12885-018-4547-7

**Published:** 2018-05-31

**Authors:** Marie-Christine Heinrich, Cosima Göbel, Martina Kluth, Christian Bernreuther, Charlotte Sauer, Cornelia Schroeder, Christina Möller-Koop, Claudia Hube-Magg, Patrick Lebok, Eike Burandt, Guido Sauter, Ronald Simon, Hartwig Huland, Markus Graefen, Hans Heinzer, Thorsten Schlomm, Asmus Heumann

**Affiliations:** 1 Institute of Pathology, University Medical Center Hamburg-Eppendorf, Martinistrasse 52, D-20246 Hamburg, Germany; 2 Institute of Neuropathology, University Medical Center Hamburg-Eppendorf, Martinistrasse 52, D-20246 Hamburg, Germany; 3 Department of Surgery, University Medical Center Hamburg-Eppendorf, Martinistrasse 52, D-20246 Hamburg, Germany; 4 Martini-Clinic, Prostate Cancer Center, University Medical Center Hamburg-Eppendorf, Martinistrasse 52, D-20246 Hamburg, Germany; 5 Department of Urology, Section for translational Prostate Cancer Center, University Medical Center Hamburg-Eppendorf, Martinistrasse 52, D-20246 Hamburg, Germany

**Keywords:** PSCA, ERG, Tissue microarray, Prostate cancer, Immunohistochemistry

## Abstract

**Background:**

Prostate Stem Cell Antigen (PSCA) is frequently expressed in prostate cancer but its exact function is unclear.

**Methods:**

To clarify contradictory findings on the prognostic role of PSCA expression, a tissue microarray containing 13,665 prostate cancers was analyzed by immunohistochemistry.

**Results:**

PSCA staining was absent in normal epithelial and stromal cells of the prostate. Membranous and cytoplasmic PSCA staining was seen in 53.7% of 9642 interpretable tumors. Staining was weak in 22.4%, moderate in 24.5% and strong in 6.8% of tumors. PSCA expression was associated with favorable pathological and clinical tumor features: Early pathological tumor stage (*p* < 0.0001), low Gleason grade (*p* < 0.0001), absence of lymph node metastasis (*p* < 0.0001), low pre-operative PSA level (*p* = 0.0118), negative surgical margin (*p* < 0.0001) and reduced PSA recurrence (*p* < 0.0001). PSCA expression was an independent predictor of prognosis in multivariate analysis (hazard ratio 0.84, *p* < 0.0001).

**Conclusions:**

The absence of statistical relationship to TMPRSS2:ERG fusion status, chromosomal deletion or high tumor cell proliferation argues against a major role of PSCA for regulation of cell cycle or genomic integrity. PSCA expression is linked to favorable prognosis. PSCA measurement is a candidate for inclusion in multi-parametric prognostic prostate cancer tests.

**Electronic supplementary material:**

The online version of this article (10.1186/s12885-018-4547-7) contains supplementary material, which is available to authorized users.

## Background

While most prostate cancers have an indolent clinical course, the disease represents the third most common cause of cancer related death in men in Western societies [1]. Gleason grade and tumor extent on biopsies, preoperative prostate-specific antigen (PSA), and clinical stage are the currently established pretreatment prognostic parameters. Although these parameters are linked to cancer aggressiveness, the distinction between indolent and aggressive prostate cancer is difficult for the individual patient. Molecular marker may enable a better prediction of prostate cancer aggressiveness in the future.

Prostate stem cell antigen (PSCA) is a protein of unknown function anchored to the cell surface. It was discovered in an attempt to identify genes up regulated in human prostate cancer [2]. Though the name implies specificity for the prostate, PSCA is expressed in several tissues: Placenta, kidney, pancreas, and bladder [3–5]. The function of PSCA has not been fully elucidated [6–9]. Experiments suggest a possible role in cell adhesion, proliferation control and cell survival [2, 10]. Evidence is accumulating that – depending on the cell type involved – PSCA can have a tumor promoting or a tumor suppressive effect [11–15]. For example, loss of PSCA was associated with poor outcome in cancer of the gallbladder and stomach [12, 16], but with improved prognosis in pancreatic adenocarcinoma, renal cell carcinoma and non-small lung cancer [17–20]. The part of PSCA in prostate cancer remains unclear. Even if most available data suggest that prostate cancer may belong to the tumors with an oncogenic function of PSCA overexpression [21–24], there are also studies that do not support such a conclusion [25] or suggest the opposite that prostate cancer aggressiveness and metastasis is driven by PSCA down regulation [26–29].

To clarify the prognostic role of PSCA expression in prostate cancer, we analyzed PSCA expression by immunohistochemistry on a large preexisting tissue micro array (TMA).

## Methods

### Patients

Radical prostatectomy specimens were from 13,660 consecutive patients operated between 1992 and 2014 at the University Medical Center Hamburg-Eppendorf (Department of Urology and Martini Clinic). In addition to the classical Gleason categories, “quantitative” Gleason grading was performed as described elsewhere [30]. Follow-up was available for 12,208 patients (Table 1). In Kaplan-Meier analysis prostate specific antigen (PSA) recurrence was defined as the time point when postoperative PSA was at least 0.2 ng/ml.

**Table 1:** Pathological and clinical data of the arrayed prostate cancers

	Study cohort on TMA (*n* = 13,660)	Biochemical relapse among category
Follow-up		
n	12,208	3017 (25%)
Mean / median	58.8 / 48.5 months	–
Age (y)		
≤50	352	61 (17%)
51–59	3335	701 (21%)
60–69	7827	1747 (22%)
≥70	2093	508 (24%)
Pretreatment PSA (ng/ml)		
< 4	1694	252 (15%)
4–10	8195	1464 (18%)
10–20	2763	847 (31%)
> 20	922	442 (48%)
pT stage (AJCC 2002)		
pT2	8861	1030 (12%)
pT3a	2984	958 (32%)
pT3b	1696	976 (58%)
pT4	71	53 (75%)
Gleason grade		
≤3 + 3	2888	236 (8%)
3 + 4	7286	1269 (17%)
3 + 4 Tertiary 5	573	133 (23%)
4 + 3	1301	594 (46%)
4 + 3 Tertiary 5	868	380 (44%)
≥4 + 4	733	404 (55%)
Nodal (pN) stage		
pN0	7904	1896 (24%)
pN+	856	524 (61%)
Surgical margin (R) status		
Negative	10,962	1939 (18%)
Positive	2649	1078 (41%)

NOTE: Numbers do not always add up to 13,660 in the different categories because of cases with missing data. Abbreviation: *AJCC* American Joint Committee on Cancer

### Immunochemistry

**Fig. 1:**
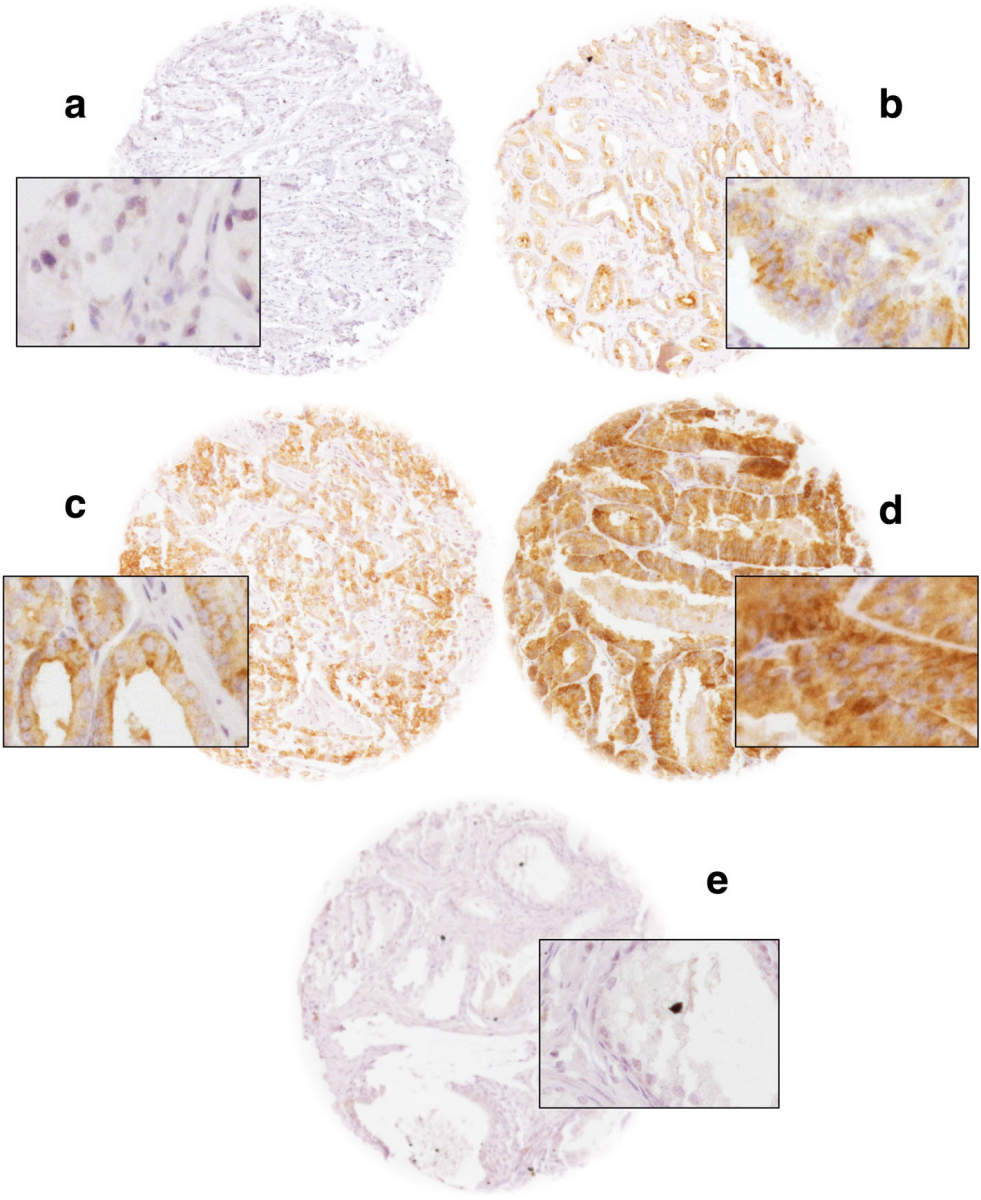
Representative images of (**a**) negative, (**b**) weak, (**c**) moderate and (**d**) strong PCSA staining in prostate cancer and (**e**) normal prostate at 100× and 400× (inset) magnification

TMAs were manufactured as described [31]. Rabbit polyclonal antibody specific for PSCA (cat#PA1–38516, Thermo scientific, dilution 1:150) was applied at 37 °C for 60 min. Bound antibody was visualized with the EnVision Kit (Dako, Glostrup, Denmark). Staining was membranous and cytoplasmic in cancer and negative in normal tissue (Fig. 1). PSCA staining was typically found in either all (100%) or none (0%) of the cells in a cancer spot. Staining intensity was semi-quantitatively assessed by visual examination of the stained slides under a microscope and grouped into four categories: Examples of negative, weak, moderate and strong staining are in Fig. 1.

### Statistics

To study association between PSCA expression and clinico-pathological variables, contingency tables were calculated and tested with the chi-square (likelihood) method. Analysis of variance and F-test was applied to find associations between PSCA expression and tumor cell proliferation. Kaplan-Meier curves were generated for PSA recurrence-free survival. Differences were checked by the log-rank test. Cox proportional hazards regression analysis was performed to test for independence and significance between pathological, molecular, and clinical variables. All calculations were done with JMP 11 (SAS Institute Inc., NC, USA).

## Results

A total of 9642 (70.6%) of TMA spots were interpretable. Non-informative cases (4023 spots; 29.4%) lacked tissue samples or unequivocal cancer tissue spots. PSCA staining was absent in glands, stromal tissue and inflammatory cells of the normal prostate. In cancers, positive PSCA staining was seen in 5581 of our 9642 (53.7%) interpretable tumors and was considered weak in 22.4%, moderate in 24.5% and strong in 6.8% of cancers. 4461 tumors (46.3%) showed no PSCA staining.

### PSCA expression and tumor phenotype

Absence of PSCA expression was linked to advanced pathological tumor stage (*p* < 0.0001), high Gleason grade (*p* < 0.0001), lymph node metastases (*p* < 0.0001), preoperative PSA level (*p* = 0.0118) and positive surgical margin (*p* < 0.0001). Data are summarized in Table 2.

**Table 2:** Association between PSCA staining and prostate cancer phenotype

	Evaluable	PSCA staining (%)				
Parameter	(N)	Negative	Weak	Moderate	Strong	*P*
Total	9642	46.3	22.4	24.5	6.8	
Tumor stage						
pT2	6003	41.5	23.6	26.9	8.0	< 0.0001
pT3a	2269	51.1	20.9	22.5	5.5	
pT3b-pT4	1326	59.7	19.5	17.0	3.8	
Gleason grade						
≤3 + 3	1713	47.0	23.0	22.8	7.2	< 0.0001
3 + 4	5275	43.4	22.8	26.5	7.4	
3 + 4 Tertiary 5	441	46.3	23.6	23.8	6.3	
4 + 3	977	50.2	21.2	21.8	6.9	
4 + 3 Tertiary 5	666	48.6	22.8	23.7	4.8	
≥4 + 4	562	61.6	17.4	17.8	3.2	
Lymph node metastasis						
N0	5873	46.0	22.4	24.4	7.2	< 0.0001
N+	674	59.9	19.1	17.7	3.3	
Preoperative PSA level (ng/ml)						
< 4	1099	48.6	19.7	25.3	6.4	0.0118
4–10	5715	44.9	23.4	24.7	7.0	
10–20	1995	46.9	21.8	24.1	7.3	
> 20	724	51.2	20.0	23.6	5.1	
Surgical margin						
Negative	7544	45.1	22.8	24.8	7.3	< 0.0001
Positive	1882	51.3	20.8	22.6	5.3	

### PSCA expression and TMPRSS2:ERG fusion

**Fig. 2:**
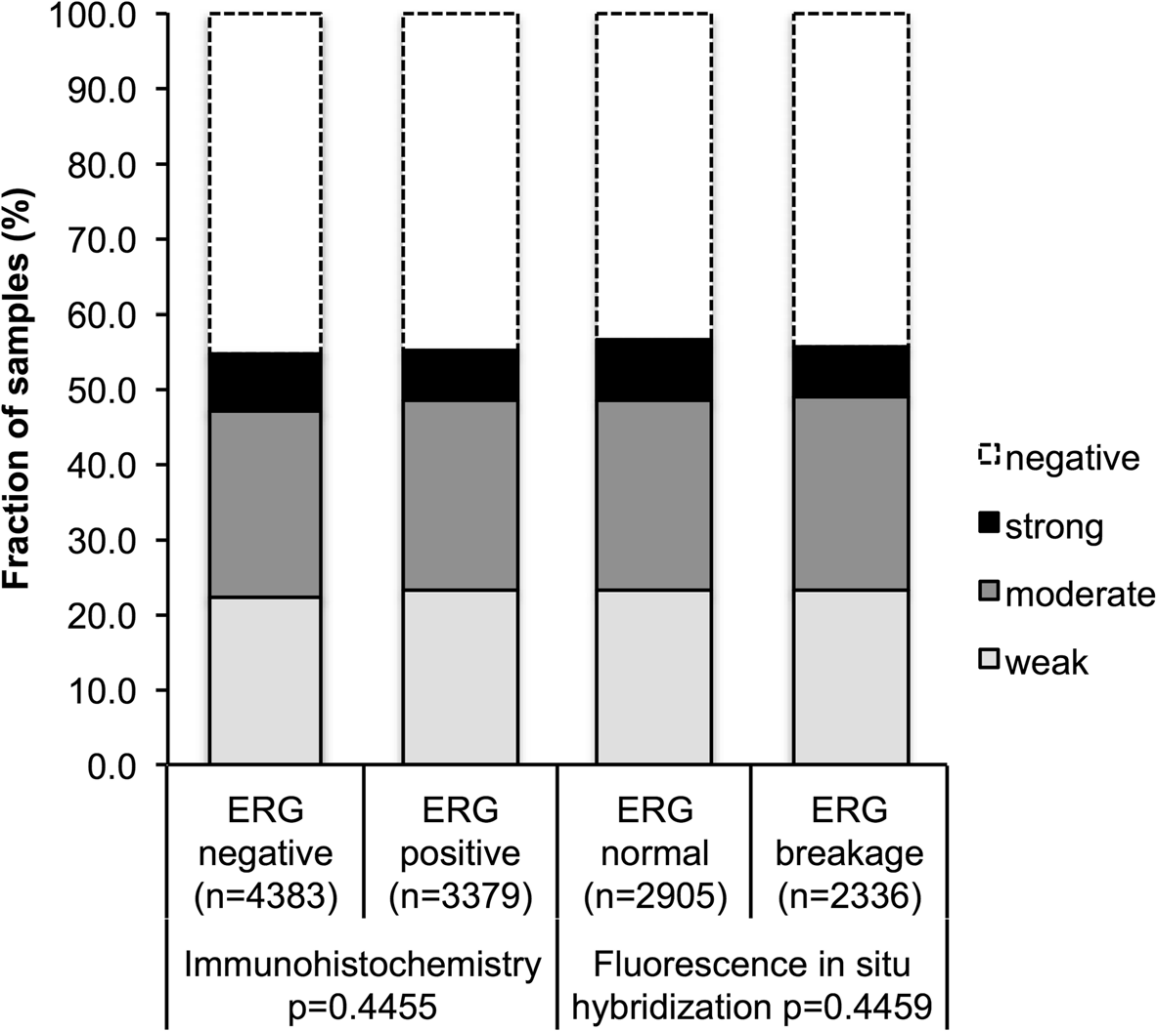
No association between PSCA staining and ERG status neither when the latter was determined by immunohistochemistry nor by fluorescence in-situ hybridization; Breakage indicates rearrangement of the ERG gene

Because TMPRSS2:ERG fusion is the predominant genetic marker in prostate cancer we analyzed its relation to PSCA expression [32]. Data on TMPRSS2:ERG fusion status obtained by FISH were available from 5241 and by immunohistochemistry (IHC) from 7762 tumors with evaluable PSCA staining. Data on both ERG FISH and IHC were available from 5042 cancers, and an identical result (ERG IHC positive and break by FISH or ERG IHC negative and missing break by FISH) was found in 95% cancers. PSCA staining did not differ significantly between ERG positive and ERG negative cancers (Fig. 2).

### Association with other key genomic deletion

**Fig. 3:**
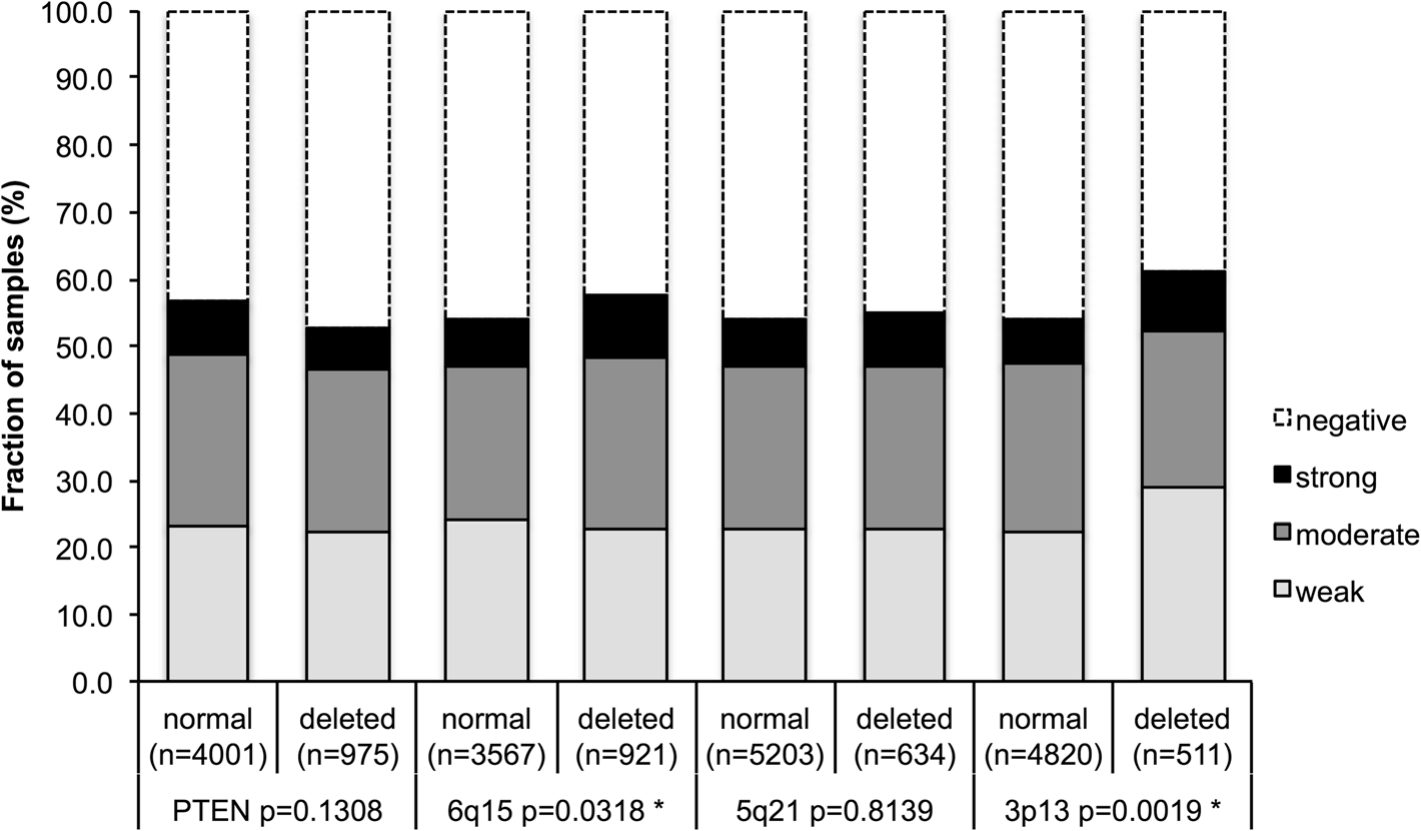
Association analysis between negative versus positive (weak + moderate + strong) PSCA expression and deletion of 10q23 (PTEN), 6q15 (*MAP3K7*), 5q21 (*CHD1*) and 3p13 *(FOXP1*). *Asterisk denotes significant *p*-value

Earlier studies had provided evidence for distinct molecular subgroups of prostate cancer defined by TMPRSS2:ERG fusion and several genomic deletions [32–37]. Therefore PSCA expression was compared with preexisting data on 10q23 (PTEN), 3p13 (FOXP1), 6q15 (MAP3K7), and 5q21 (CHD1) deletion. PSCA expression did not differ notably between cancers with and without these deletions with the exception of marginal association of positive PSCA expression and 6q15- (*p* = 0.0318) respective 3p13- deletion (*p* = 0.0019, Fig. 3).

### Tumor cell proliferation

No association was found between PSCA staining and tumor cell proliferation as measured by Ki67 labeling index (*p* = 0.2211), neither in all cancers nor in subsets of ERG negative or ERG positive cancer, or in tumor subsets with identical Gleason score (*p* > 0.05; data not shown).

### Association with PSA recurrence

**Fig. 4:**
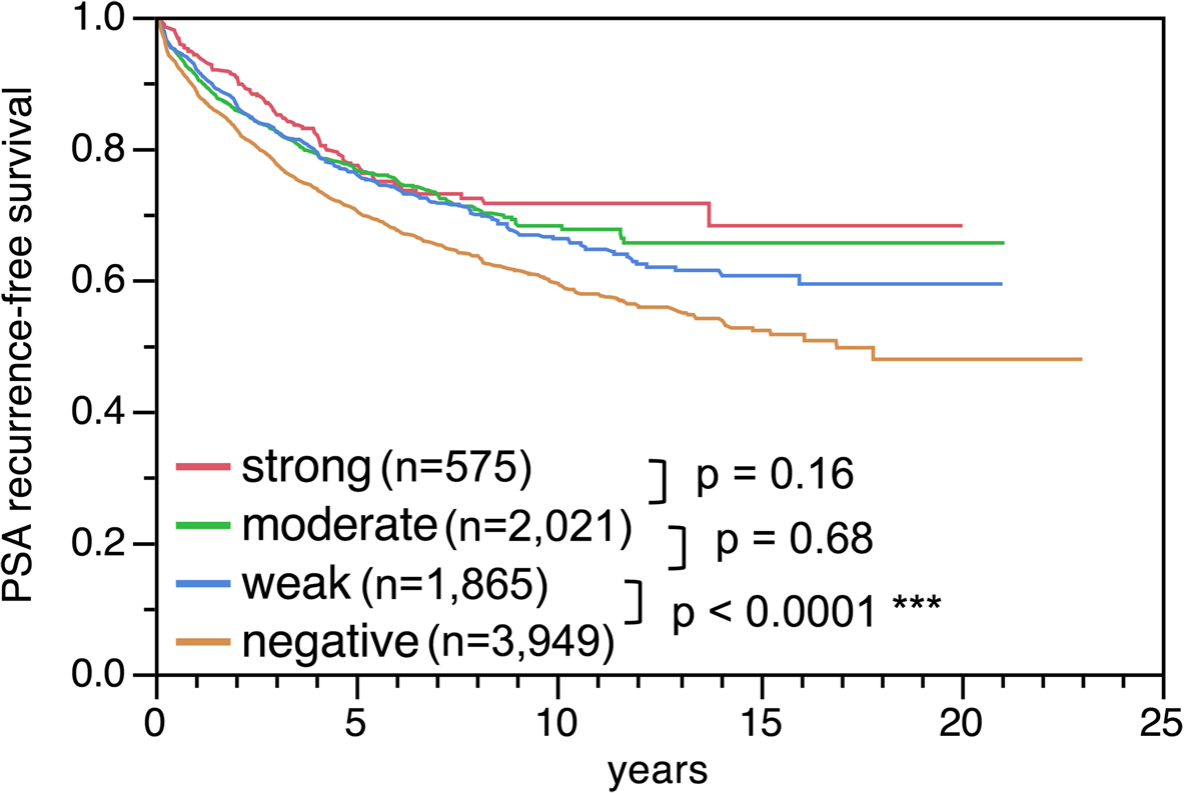
Kaplan-Meier analysis of prostate specific antigen (PSA) recurrence after radical prostatectomy and negative versus weak, moderate and strong PSCA expression. *Asterisk denotes significant *p*-value

**Fig. 5:**
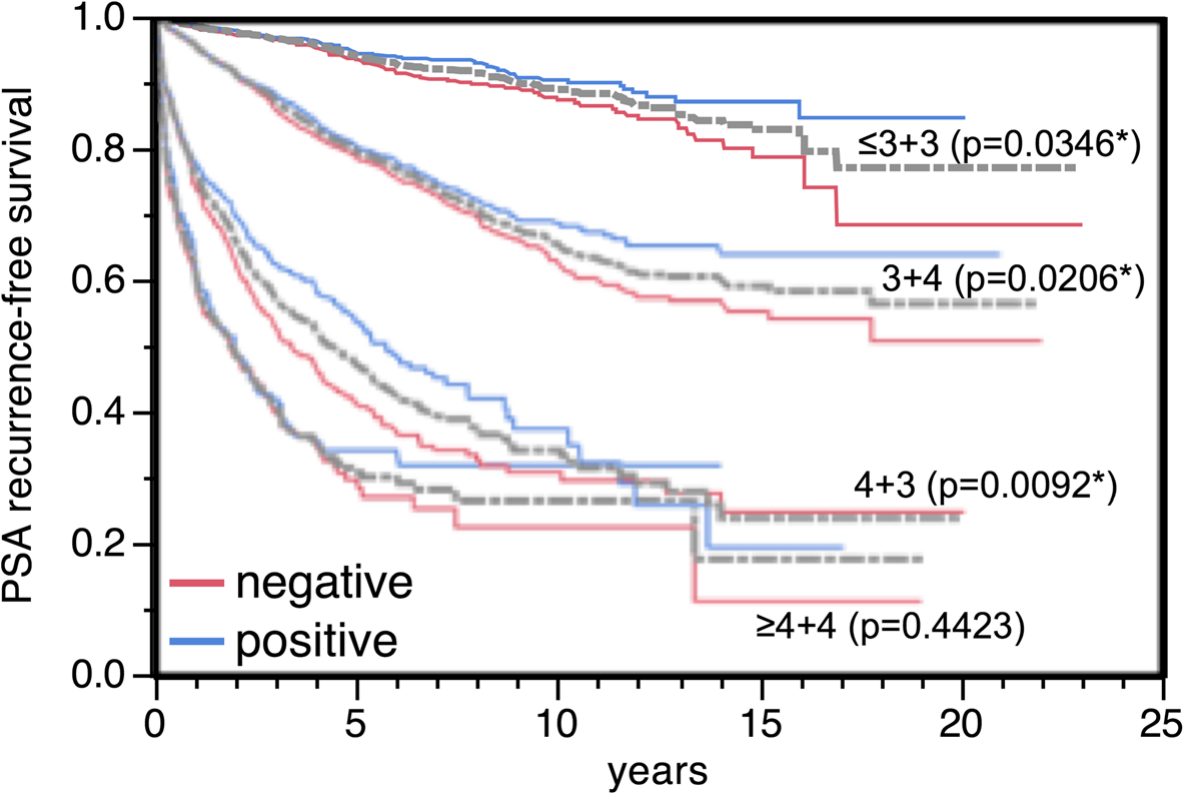
Kaplan-Meier plot of PSA recurrence-free survival and negative or positive (weak + moderate + strong) PSCA expression stratified for Gleason grade (≤3 + 3, *n* = 1535; 3 + 4, *n* = 3430; 4 + 3, *n* = 984; ≥4 + 4, *n* = 323). *Asterisk denotes significant *p*-value

Follow-up data were available from 8410 patients with interpretable PSCA staining. Tumors with negative PSCA staining showed a significantly shortened PSA recurrence-free interval compared with positively stained cancers (*p* < 0.0001, Fig. 4). This holds true for the subgroup of ERG fusion negative and positive cancer (data not shown). In further analysis weak, moderate and strong stained tumors were grouped as positive. PSCA expression provided additional prognostic impact in most subsets of cancer with identical classical Gleason grade group (*p* = 0.0346 for ≤3 + 3, *p* = 0.0206 for 3 + 4, *p* = 0.0092 for 4 + 3 and *p* = 0.4423 for ≥4 + 4, Fig. 5). However, the prognostic impact of PSCA expression was lost in subgroups with comparable quantitative Gleason scores (Additional file 1: Figure S1).

### Multivariate analysis

Four different multivariate scenarios were used to simulate clinical decisions (Table 3). Scenario 1 evaluated the preoperatively available parameters: Preoperative Gleason grade obtained on the original biopsy, clinical tumor stage (cT stage) and preoperative PSA together with the postoperatively obtained PSCA expression. In scenario 2, the radical prostatectomy Gleason grade replaced the biopsy Gleason grade, while in scenario 3 pathological (pT) stage and surgical margin (R) status replaced cT stage. In scenario 4, the lymph node (pN) stage is added. Overall, PSCA expression proved to be an independent favorable prognostic parameter. The Cox hazard ratio for PSA recurrence-free survival after radical prostatectomy between weak and negative PSCA expression varied from 0.84 to 0.93 and was significant in scenario 1 and 2 (Table 3).

**Table 3:** Hazard ratios of established prognostic parameters and PSCA expression in prostate cancer

Scenario	1	2	3	4
Analyzable (N)	8334	8454	8570	5801
Gleason grade biopsy				
3 + 4 vs. ≤3 + 3	1.94***			
4 + 3 vs. 3 + 4	1.63***			
≥4 + 4 vs. 4 + 3	1.39***			
cT stage				
T2a vs. T1c	1.53***	1.47***		
T3a vs. T2c	0.65*	1.07		
Preoperative PSA level				
4–10 vs. < 4	1.25*	1.21*	1.11	1.14
10–20 vs. 4–10	1.58***	1.41***	1.25***	1.16*
> 20 vs. 10–20	1.67***	1.47***	1.23*	1.22*
PSCA expression				
Positive vs. negative	0.84***	0.86**	0.93	0.93
Gleason grade prostatectomy				
3 + 4 vs. ≤3 + 3		2.91***	2.39***	2.30***
4 + 3 vs. 3 + 4		2.72***	2.24***	2.05***
≥4 + 4 vs. 4 + 3		1.75***	1.25*	1.21*
pT stage				
T3a vs. T2			1.94***	1.94***
T3b vs. T3a			1.73***	1.52***
T4 vs. T3b			1.20	1.24
Surgical margin (R) status				
R1 vs. R0			1.40***	1.18*
Nodal (pN) stage				
N+ vs. N0				1.56***

Scenario 1 combines preoperatively available parameter (preoperative Gleason grade obtained on the original biopsy, clinical tumor (cT) stage, and preoperative PSA) with the postoperative PSCA expression. In scenario 2 the biopsy Gleason is replaced by the Gleason grade obtained on radical prostatectomy. In scenario 3 cT stage is superseded by pathological tumor (pT) stage and surgical margin (R) status. In scenario 4 the lymph node (pN) stage is added. Asterisk indicate significance level: * *p* ≤ 0.05, ** *p* ≤ 0.001, and *** *p* ≤ 0.0001

## Discussion

This study demonstrates that PSCA expression is significantly associated with favorable tumor phenotype and a reduced risk for PSA recurrence. A total of 54% of prostate cancers showed detectable PSCA expression in our IHC study. This is in the range of two other studies reporting in 88% of 126 [2] or 48% of 233 patients [4] IHC positivity. These IHC findings are not contradictory to further studies describing at least a low level PSCA expression in all prostate cancers utilizing polymerase chain reaction [5, 23]. It is well known that IHC negativity does not reflect the absence of expression but rather that a certain threshold for detection is not reached. The threshold of detection is greatly influenced by the IHC protocol [38]. Although Ross et al. [4] and Reiter et al. [2] used different antibodies and protocols; they obviously resulted in a comparable sensitivity as in our study. We consider our protocol suited for studying the prognostic impact of PSCA expression as the selected conditions enable a distinction of cancers with high and low levels of PSCA expression. Given that normal prostate epithelial cells usually did not stain for PSCA, we assume that PSCA up-regulation had occurred in a fraction of prostate cancers. The link between cancer specific up-regulation of a protein and better prognosis is uncommon and argues for a “protective” or tumor suppressive role. Our outcomes are in concordance with data from Larkin et al. [27], also reporting a link between elevated PSCA expression and favorable clinical course. A tumor suppressive role of high level PSCA expression is also supported from cell line models. For example, functional analysis of cell lines from gastric and gallbladder cancer demonstrated that forced overexpression of PSCA hampered cell proliferation [12, 16].

It is noteworthy that several investigators reported contrary results. IHC with various and partly custom made antibodies to conventional large sections of 40 [22] and 112 [5] prostate cancers revealed associations with high Gleason score, advanced stage and castration resistant disease. Also in TMA studies including 114 [23] and 246 [24] prostate cancers the authors reported associations between strong PSCA expression and high Gleason score. However, another TMA study on 64 prostate cancers could not confirm these findings [25]. We cannot explain the discrepancy between these studies and our data obtained on almost 10,000 successfully analyzed carcinoma.

The mechanism for a tumor suppressive function of PSCA is unknown. In our study, we compared the expression of PSCA with molecular attributes associated with genomic instability, chromosomal deletion and tumor cell proliferation [37]. We found previously that features with a role in cell cycle control (p16 [39] or APE1 [40]) were significantly associated with a high Ki67 labeling index. Molecular attributes linked to genomic instability (MSH6/PMS2/MLH1 [41], ELAV1 [42], or HOOK3 [43]) were found to be associated with chromosomal deletion. The lack of clear-cut association with these features argues against a relevant impact of PSCA on cell proliferation control and development of deletion in prostate cancer. This is in contrast to one experimental study, suggesting an accelerating effect of PSCA loss on cell proliferation in a gastric cancer cell line [16]. That PSCA expression was completely unrelated to TMPRSS2:ERG fusion further demonstrates that PSCA is not significantly affected by any of the hundreds of genes that are deregulated in ERG fusion positive prostate cancer [44–47].

PSCA expression was significantly associated with favorable patient outcome in our cohort. A possible clinical relevance of this finding is supported by its statistical independence of classical prognostic markers, especially in a pre-operative disease state. However, in comparison with established features such as the Gleason score, the impact of PSCA expression on patient outcome was rather small. If traditional prognostic Gleason groups were used, a small prognostic impact was still found in Gleason 3 + 4 (*p* = 0.0206) or Gleason 4 + 3 (*p* = 0.0092). However, if these subgroups were further differentiated according to the fraction of Gleason 4 (quantitative Gleason grading [30]) these PSCA associated prognostic differences vanished. This further illustrates the high bar that molecular characteristics have to overcome if compared with optimized morphologic analysis.

## Conclusions

PSCA expression is a statistically independent predictor of favorable prognosis in prostate cancer. Although its prognostic impact per se is not very strong, PSCA expression analysis could be considered for inclusion in multi-parametric prognostic tests to distinguish prostate cancers with need for radical therapy.

## Additional file

Additional file 1: **Figure S1.** Kaplan-Meier plot of prostate specific antigen (PSA) recurrence and PSCA expression stratified for quantitative Gleason grade. Note the different time scale for Gleason Tertiary 5 grades. (DOC 2467 kb)

## Abbreviations

AR: Androgen receptor
CHD1: Chromodomain-Helicase-DNA-Binding Protein 1
ELAV1: Embryonic Lethal, Abnormal Vision, Drosophila)-Like 1
FISH: Fluorescence in-situ hybridization
FOXP1: Forkhead box protein P1
HOOK3: Hook Microtubule Tethering Protein 3
IHC: Immunohistochemistry
MAP3K: Mitogen-Activated Protein Kinase Kinase Kinase 7
PSCA: Prostate stem cell antigen
PSA: Prostate specific antigen
PTEN: Phosphatase and tensin homolog
RPE: Radical prostatectomy
TMPRSS2: Transmembrane protease, serine 2
ERG: ETS-related gene fusion
TMA: Tissue microarray
