# Supplementary material for: PSCA expression is associated with favorable tumor features and reduced PSA recurrence in operated prostate cancer
Source: BMC Cancer. 2018 May 31;18:612. doi: 10.1186/s12885-018-4547-7 (PMC5984312; doi:10.1186/s12885-018-4547-7)
Supplement: Supplementary file 1 — Figure S1. Kaplan-Meier plot of prostate specific antigen (PSA) recurrence and PSCA expression stratified for quantitative Gleason grade. Note the different time scale for Gleason Tertiary 5 grades. (DOC 2467 kb) [file 12885_2018_4547_MOESM1_ESM.doc]

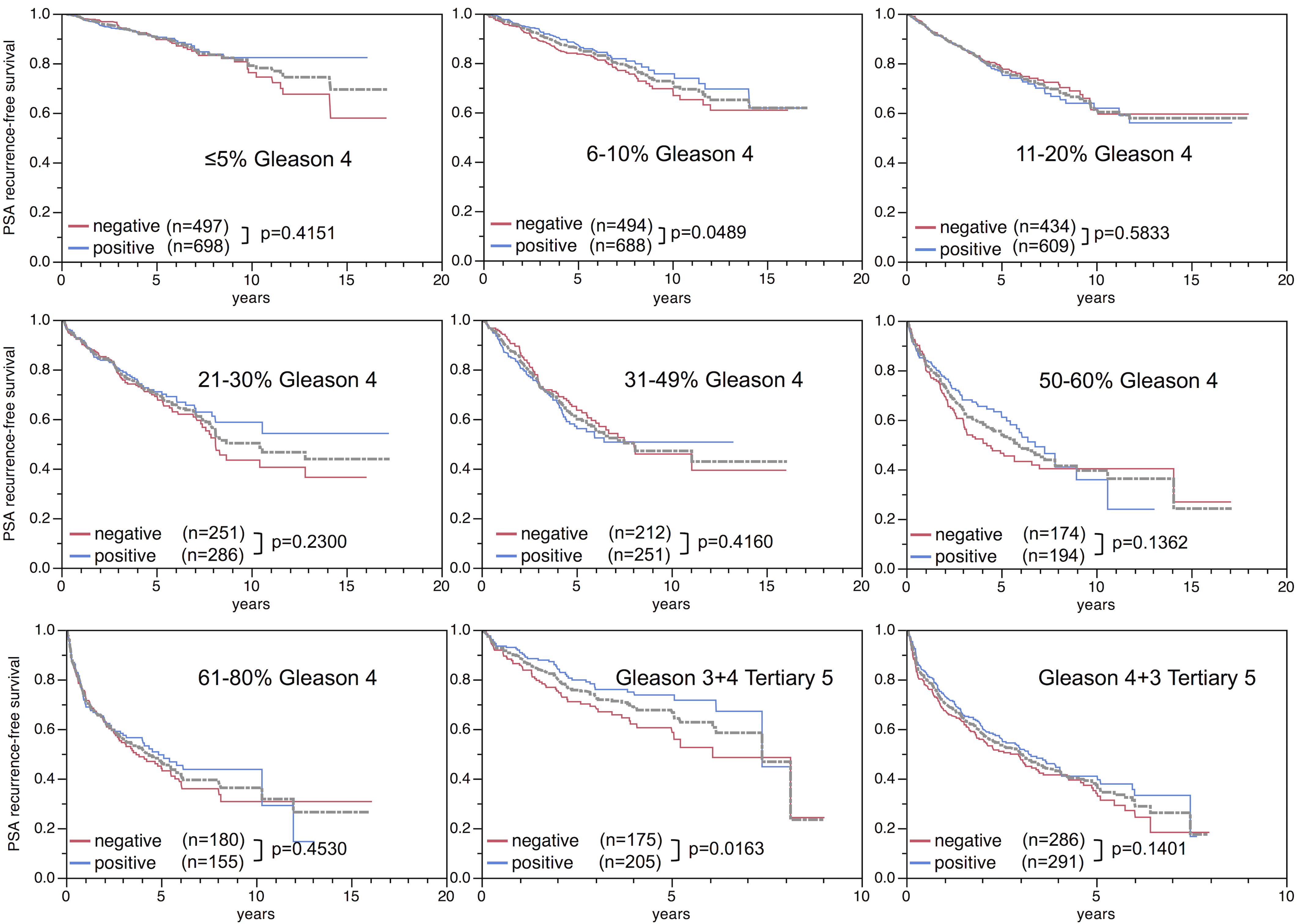


**Figure S1.** Kaplan-Meier plot of prostate specific antigen (PSA) recurrence and PSCA expression stratified for quantitative Gleason grade. Note the different time scale for Gleason Tertiary 5 grades.
